# Ultrasound localization microscopy and functional ultrasound imaging reveal atypical features of the trigeminal ganglion vasculature

**DOI:** 10.1038/s42003-022-03273-4

**Published:** 2022-04-07

**Authors:** Annabelle Réaux-Le-Goazigo, Benoit Beliard, Lauriane Delay, Line Rahal, Julien Claron, Noémi Renaudin, Isabelle Rivals, Miguel Thibaut, Mohamed Nouhoum, Thomas Deffieux, Mickael Tanter, Sophie Pezet

**Affiliations:** 1 Sorbonne Université, INSERM, CNRS, Institut de la vision, 17 rue Moreau, 75012 Paris, France; 2 Physics for Medicine Paris, Inserm, ESPCI Paris, CNRS, PSL Research University, 17 rue Moreau, 75012 Paris, France; 3 Equipe de Statistique Appliquée, ESPCI Paris, PSL Research University, UMRS 1158, 10 rue Vauquelin, 75005 Paris, France; 4 Iconeus, 27 Rue du Faubourg Saint-Jacques, 75014 Paris, France

**Keywords:** Neuroscience, Biophysics

## Abstract

The functional imaging within the trigeminal ganglion (TG) is highly challenging due to its small size and deep localization. This study combined a methodological framework able to dive into the rat trigeminal nociceptive system by jointly providing 1) imaging of the TG blood vasculature at microscopic resolution, and 2) the measurement of hemodynamic responses evoked by orofacial stimulations in anesthetized rats. Despite the small number of sensory neurons within the TG, functional ultrasound imaging was able to image and quantify a strong and highly localized hemodynamic response in the ipsilateral TG, evoked not only by mechanical or chemical stimulations of corneal nociceptive fibers, but also by cutaneous mechanical stimulations of the ophthalmic and maxillary orofacial regions using a von Frey hair. The in vivo quantitative imaging of the TG’s vasculature using ultrasound localization microscopy combined with *in toto* labelling reveals particular features of the vascularization of the area containing the sensory neurons, that are likely the origin of this strong vaso-trigeminal response. This innovative imaging approach opens the path for future studies on the mechanisms underlying changes in trigeminal local blood flow and evoked hemodynamic responses, key mechanisms for the understanding and treatment of debilitating trigeminal pain conditions.

## Abstract (web summary)

Visualisation of rat trigeminal ganglia activation during ophthalmic or maxillary nociceptive stimulations shows atypical tortuous vascularisation and a somatotopic hemodynamic response.

## Introduction

The trigeminal ganglion (TG) contains the cell body of the primary sensory neurons from the ophthalmic (V1), the maxillary (V2), and the mandibular (V3) nerves. These sensory neurons are highly specialized, as they detect and respond to a variety of chemical, mechanical, and thermal stimuli applied on these regions.

Because the TG is relatively small and localized in Meckel’s trigeminal cave in both human and rodents, only a limited number of studies were able to perform functional neuroimaging studies. While Bererra’s and Borsook’s teams published seminal works on the existence of a vascular response in the human TG^1–4^, only one preclinical contrast MRI study imaged macrophage infiltration in the mouse TG using ultrasmall superparamagnetic iron oxide nanoparticle contrast in a model of alkali burn cornea^5^. But, to the best of our knowledge, dynamic functional imaging of the TG was never performed in rodents due to the difficulties to access the TG. Preclinical studies investigating the physiological activity within the TG of rodents are classically based on electrophysiological recordings of single and/or clusters of neurons^6,7^, as well as immunohistochemical staining using indirect markers of neuronal activation (see ref. ^8^ for review). Despite the cellular resolution of these surrogates, this approach lacks the ability to follow the dynamics of these neuronal changes. Recently, the visualization of trigeminal sensory neuron activities in response to orofacial stimuli was reported ex vivo using either voltage-sensitive dye approach in decerebrated animals^9^ or calcium imaging in GCaMP6 mouse line^10,11^. However, these highly invasive experimental paradigms require the decerebration of the animal, and therefore the disconnection between TG and the CNS.

Functional ultrasound (fUS) imaging is a relatively new versatile neuroimaging modality that allows imaging and measurement of cerebral blood volume in both human^12,13^, non-human primates^14^ and rodents^15–19^ with excellent spatial (100–300 µm) and temporal resolutions (down to 20 ms). One of its biggest advantages is its high sensitivity compared to fMRI^20–22^. Indeed, during a task, the locally increased neuronal activity due to the neurovascular coupling leads to a hemodynamic response^23^. The direct link between fUS signal and neuronal activity was recently described, as well as the hemodynamic response function^21,24^. In the past, fUS imaging has proven sensitive enough to measure the cortical hemodynamic changes induced by optogenetic stimulations^22,25^, sensory^18,25^, olfactory^26^, and visual^27,28^ stimuli in anesthetized animals, as well as auditory stimuli^29^ and motor tasks^14–16^ in awake animals. Interestingly, fUS can be coupled on the same device with another emerging modality, Ultrasound Localization Microscopy (ULM), enabling the observation of the brain vascular anatomy and blood flow up to microscopic resolution both in rodents^30^ and humans^31^.

The corneal trigeminal system is particularly interesting as the cornea is the most densely innervated tissue in the body^8^ whose nerve terminals are directly accessible for stimulation. Moreover, the cornea is exclusively innervated by unmyelinated C- and thinly myelinated A delta fibers, including mechano-nociceptors that are triggered by noxious mechanical stimulation, polymodal nociceptors that are excited by mechanical, chemical, and thermal stimuli, and cold thermoreceptors that are activated by cooling^8,32,33^.

Taking advantage of the high sensitivity of fUS imaging, this study had several main objectives: first to localize the TG in anesthetized rats, second to measure the velocity of blood flow in the TG using ULM, and third to detect and measure the functional activation in the TG induced by peripheral stimulations of various orofacial trigeminal divisions (ophthalmic V1 and maxillary V2). We provide the first proof of concept of imaging the rat’s TG, with a detection of local blood flow at a microscopic scale, and of the measurement of the hemodynamic responses evoked by the activation of trigeminal nociceptors in anesthetized animals. Our results bring forward an innovative approach to study the TG’s evoked hemodynamic responses, a key element for deciphering the mechanisms of trigeminal sensitization and concomitant pain characteristic of trigeminal pathologies.

## Results

### Localization/imaging of the rat trigeminal ganglia using ultrafast Doppler imaging

**Fig. 1:**
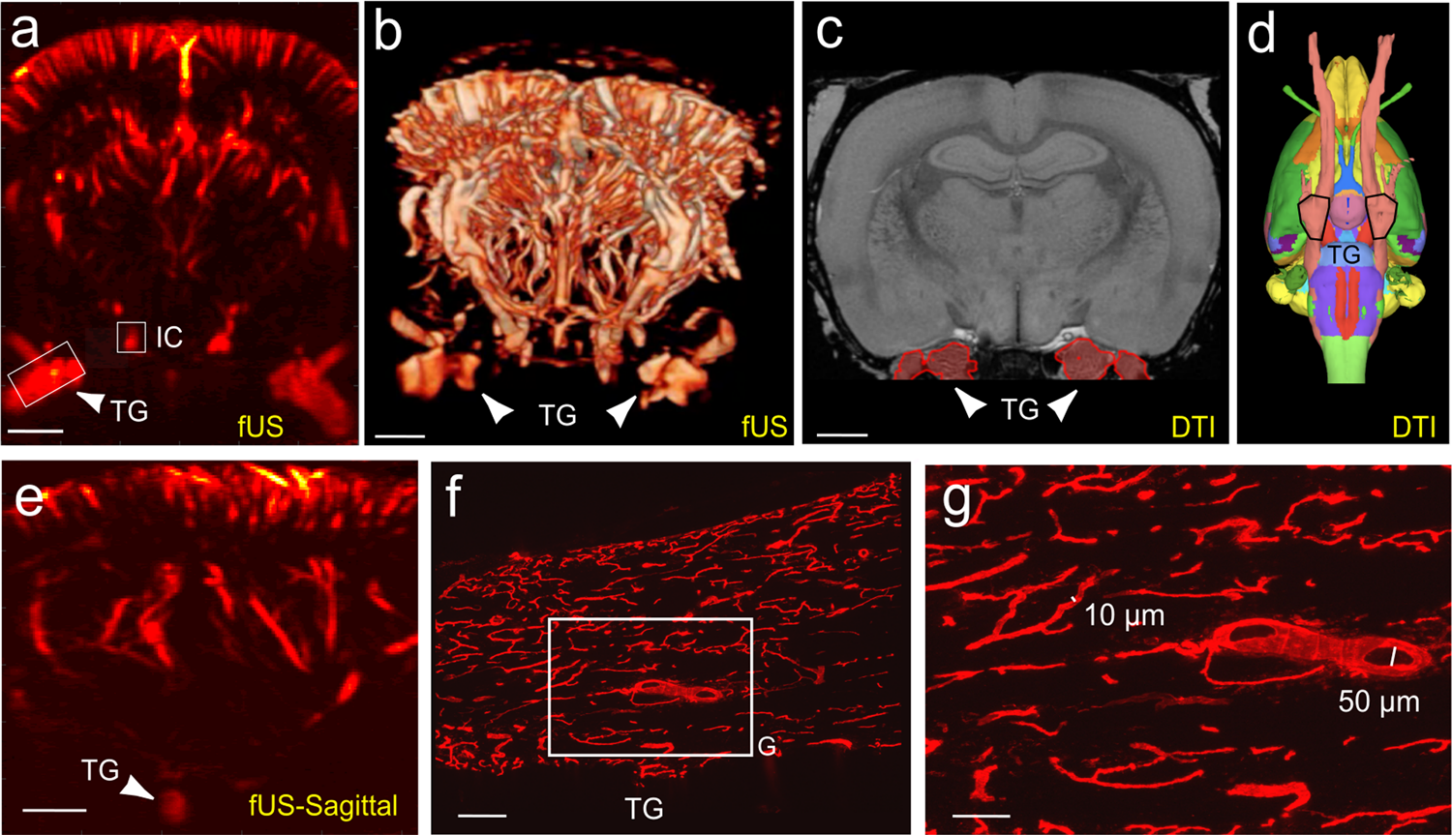
Anatomical localization and vascularization of the rat trigeminal ganglia. **a**, **e** fUS imaging through the whole brain depth reveals the vascularization and localization of the rat TG in a coronal plane (**a**) or sagittal plane (**e**). **b** Capture of the 3D tomographic scan detailed in Supplementary Movie 1, illustrating the TGs seen in 3D (white arrows). **c**, **d** Localization of the trigeminal ganglion according to Waxholm’s tractography atlas (https://scalablebrainatlas.incf.org/rat/PLCJB14, i.e., defined using fiber tracks^34–36^), observed coronally (**c**, white arrows) or in 3D (**d**, black line surrounding of the ganglia) medio-lateral, at the base of the brain. **f**, **g** In toto vascular staining in the rat TG showing that this structure is richly vascularized by a dense network of both thin (10 µm diameter) blood vessels, but also large blood vessels (50 μm diameter). DiI was used to stain the endothelial cell membrane lining the blood vessels, as previously described^60^. IC internal carotid. Scale bars = **a**, **b**, **c**: 2 mm, **e**: 1.5 mm, **f**: 200 μm, **g**: 100 μm. Images of the Waxholm’s tractography atlas, which are distributed under the Creative Commons Attribute Non-Commercial Share-Alike license, were copied, without any modification.

Taking into account that TG is a deep structure, we imaged much deeper under the brain as compared to previous studies in anaesthetized rodents^18,19^. Despite the signal attenuation due to the depth of imaging (15 mm), two-dimension scans revealed two bilateral longitudinal structures detected between the antero-posterior coordinates Bregma −3.3 and Bregma −5.0 mm (Fig. 1a, b, f). In addition to two-dimension scans (Fig. 1a, e), three-dimension scans (Fig. 1b, Supplementary Movie 1) localized the structure precisely with respect to the location of the brain’s vasculature.

Comparison with the Waxholm tractography atlas (https://scalablebrainatlas.incf.org/rat/PLCJB14,^34–36^) confirmed that these bilateral structures are located under the brain at the same antero-posterior coordinates and laterality as the TG they described using tractography of peripheral fibers (Fig. 1c, Supplementary Fig. 1).

**Fig. 2:**
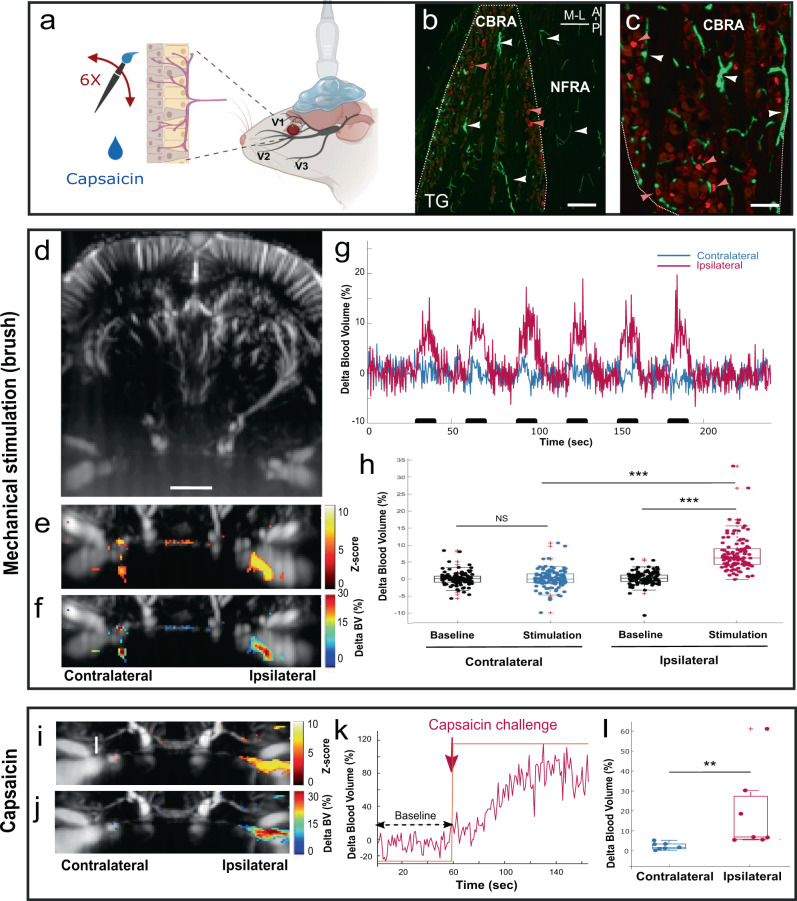
Mechanical and chemical activation of corneal mechano- and polymodal nociceptors induce a functional hyperaemia in the ipsilateral trigeminal ganglion. **a** Schematic presenting the corneal afferents (C and A delta fibers) and fUS imaging of the TG. **b**, **c** Double staining CGRP (red)/vascular in toto staining (DiI, Green) showing that the TG is richly vascularized, especially in the ‘Cell Body Rich Area’ (CBRA), delineated by a dashed white line (**b**, **c**). The vascularization is on the contrary sparse in the ‘Nerve Fibers Rich Area’ (NFRA). Red arrowheads point at CGRP-positive sensory neurons located in the near proximity of stained blood vessels (white arrowheads). Panel **c** displays a high-power magnification taken in another animal. **d**–**h** Hemodynamic responses in the TG induced by mechanical stimulations of the cornea. **d** Example of Doppler image of the brain and trigeminal ganglion, imaged through an acute craniotomy, **e** map of Z-score and **f**: delta blood volume (BV) change in a representative example, illustrating the response in the medio-lateral part of the TG. Panel **g** shows the spatially averaged temporal changes in TG’s blood volume in the ipsilateral (red) and contralateral (blue) region of interest delineated by the Z-score. **h** Quantification of the changes in TG blood volume in 18 trains of stimulations, (obtained from 8 animals) reveals a significant hyperhaemia in the ipsilateral, but not contralateral TG. **i**–**l** Phasic hemodynamic response in the TG induced by activation of the TRPV1 polymodal corneal nociceptors by topical administration of capsaicin (10 μM). **i**–**k** Example of Z-score map (**i**), delta BV (**j**), and temporal changes of the TG’s blood volume (**k**) in the ipsilateral region of interest delineated by the Z-score in a representative animal. **l** Quantification of the changes in TG delta BV (*N* = 7 animals). For all panels, the region of interest (ROI) drawn in the contralateral side was the symmetrical ROI of the ipsilateral site. In **h** and **l**: results are presented an overlay of both boxplots (median, first and third quartiles) and individual values. The red crosses are outliers. *p < 0.05, **p < 0.01, ***p < 0.001. Scale bars = **b**: 200 μm, **c**: 100 μm, **d**, **e**, **f**, **i**, **j**: 2 mm. Panel **b**: A-P and M-L indicate the antero-posterior and the medio-lateral directions, respectively. They also apply to the panel **c**. The panel **a** was created with Biorender.com.

It is known that Doppler signals in deep structures are reduced due to attenuation. Since the Doppler signal of the TG was visible at this depth despite the attenuation, we hypothesized that the TG might be highly vascularized. This hypothesis was confirmed by comparing the Doppler signal between the TG and the internal carotid (Fig. 1a). We found that the blood volume measured in the TG (without correction for the attenuation) represents 44.0 ± 1.4% of that of the internal carotid. This hypothesis was finally also verified using in toto *(*DiI) labelling of the TG vessels in fixed tissues. This histological approach revealed a high density of DiI labelled vessels, which exhibited a tortuous morphology (Fig. 1f, g). Altogether, ex vivo staining and in vivo fUS imaging data support the idea that the TG receives an important blood supply and is richly vascularized, especially in the area containing the cell bodies (CBRA, Fig. 2b, c).

### Corneal nociceptor stimulations induce functional hyperaemia in the ipsilateral trigeminal ganglion

Our anatomical study confirmed a high density of blood vessels stained in vivo using DiI in close interaction with small and medium diameter CGRP + primary sensory neurons in the ophthalmic branch of the TG (Fig. 2b, c). This anatomical organization of trigeminal CGRP nociceptors is suspected by several authors^37^ to play a role in orchestrating the hemodynamic response to corneal stimulation. Due to both the rich vascularization of the TG and the high sensitivity of ultrafast Doppler to slight blood volume (BV) changes linked to the stimulus^14^ at the pixel level^20,29^, we postulated that fUS imaging could provide the first evidence of hemodynamic responses evoked by corneal stimulations in the ophthalmic division of the TG. To support this hypothesis, we used stimulations of the cornea as a system model, as the cornea is richly innervated by C and A delta fibers (Fig. 2a) and has the advantage to be easily activated by external mechanical and chemical stimulations.

Despite the small number of corneal neurons^38^, repetitive mechanical stimulations of the cornea induced strong and reproducible hemodynamic responses in the ipsilateral TG (Fig. 2e–g, red line, 7.3% ∆BV increase, *p* = 3.3 × 10^−22^), but not in the contralateral TG (blue line, 0.02% increase, *p* = 0.92), the difference between the ipsilateral and contralateral delta blood volumes during stimulation being also highly significant (7.3% ∆BV increase, Fig. 2h, *p* = 1.7 × 10^−23^). Supplementary Movie 2 shows a typical experiment with a visualization in real time of changes in BV during repeated mechanical corneal stimulations. Furthermore, the significant hemodynamic response remained stable during the duration of every single corneal stimulation, suggesting the absence of desensitization in both the peripheral C-fiber activation and the mechanisms underlying the neurovascular coupling.

Finally, the activation of TRPV1 polymodal corneal nociceptors using the TRPV1 ligand capsaicin (10 μM) induced a phasic and robust increased blood volume (18.9% increase, *p* = 9.3 × 10^−3^) in the ipsilateral TG as compared to the contralateral TG (Fig. 2i–l). The BV increase lasted during all the time of exposure to the drug (1 min, Fig. 2k).

### Cutaneous mechanical stimulations using a von Frey hair in the ophthalmic and maxillary territories induce different segregated areas of hyperaemia in the ipsilateral TG

The previous experiments (corneal stimulations) were performed on an acute craniotomy preparation which presents not only several advantages, such as its simplicity for terminal experiments, but also several drawbacks, such as the requirement of analgesia in the anesthetic used (here ketamine), the limited physiological aspect of this preparation and finally possible spurious local cortical activation during the imaging session. While we and others are routinely using these acute preparations^18,19,28^, two other physiological preparations are also routinely used: skull thinning^19,39,40^, which is only temporary since the skull regrows, or preparation of a chronic window^17,25,41,42^.

**Fig. 3:**
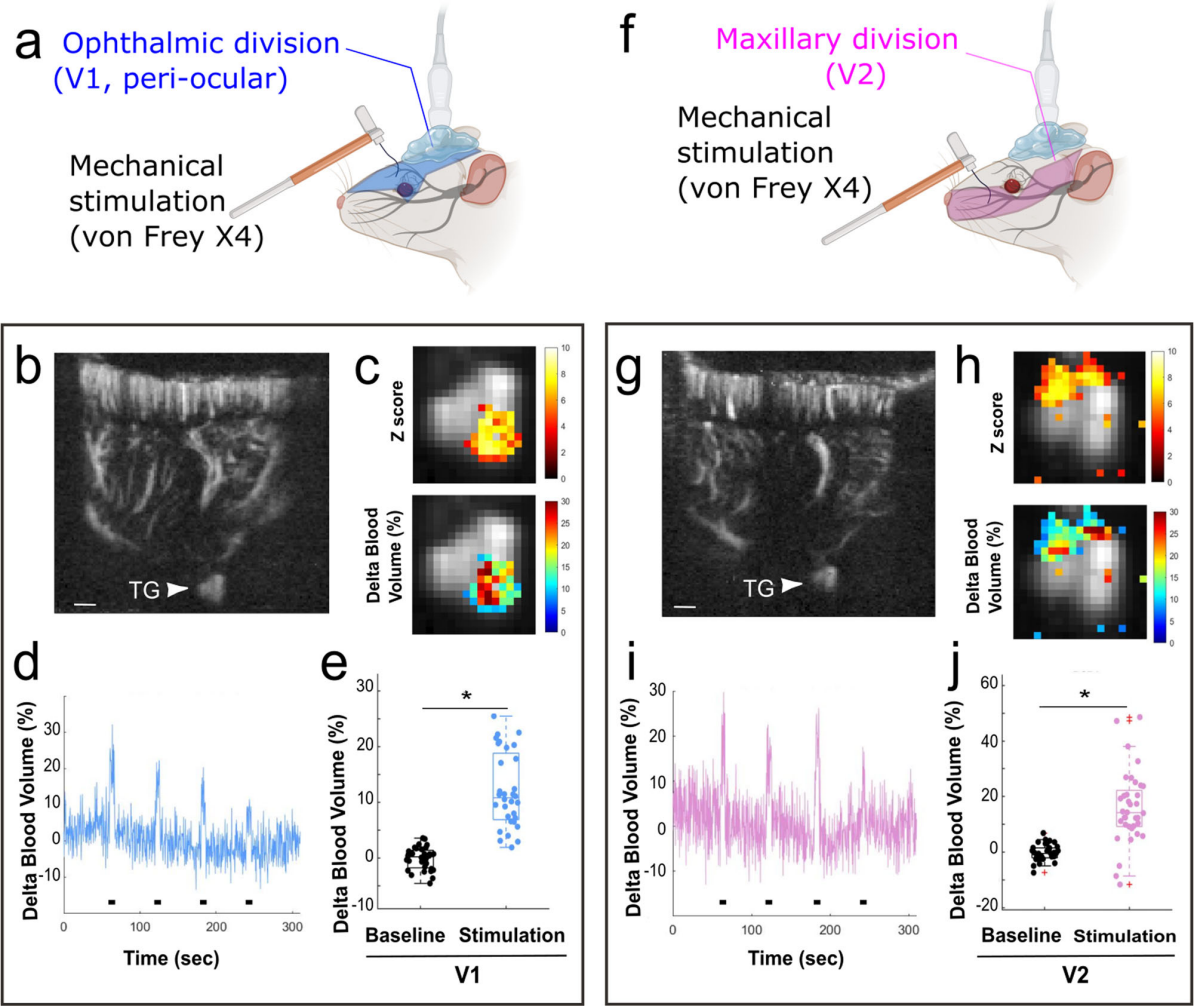
Static mechanical stimulations of the ophthalmic and maxillary areas using a von Frey hair induced a functional hyperaemia in the ipsilateral trigeminal ganglion. **a**, **f** Diagram representing the stimulated trigeminal subdivision (**a**: Ophthalmic, **f**: Maxillary). Due to the light anesthesia required for these experiments, imaging was performed through a chronically implanted window, using isoflurane as anesthetic. Panels **b**–**d** and **g**–**i** show representative results obtained in the same animal, using stimulations of the ophthalmic (V1, **b**–**d**) or maxillary (V2, **g**–**i**) divisions. Due to the natural orientation of the TG in the cranial cavity, but also the different lateralities of the V1 and V2 subdivisions, sagittal planes of imaging were used, at the following coordinates: lateral = 6 mm, rotation = 8° for the V1 and lateral = 5 mm, rotation = 12° for V2. For each half panel, **b** and **g** show the Doppler image of the plane imaged, with the brain and the TG (indicated by an arrowhead). Panels **c**, **d** and **h**, **i** show examples of Z-score maps (**c**, **h** top), delta BV maps (**c**, **h** bottom) and the average temporal changes of the delta BV (**d**, **i**) over the region of interest delineated by the Z-score. **e**, **j** Quantification of the changes in delta BV (**e**: *N* = 30 stimulations in *N* = 3 animals; **i**: *N* = 36 stimulations in *N* = 3 animals). Consistent with previous observations of the TG’s somatotopic organization, the stimulation of the ophthalmic and maxillary branches evoked responses in different parts of the TG (dorsal for the maxillary, ventral for the ophthalmic). In **e** and **j**: results are presented an overlay of both boxplots (median, first, and third quartiles) and individual values. The red crosses are outliers. *p < 0.05. Scale bars: **b**–**g**: 100 μm. Panels **a** and **f** were created with Biorender.com.

In order to image and to quantify the evoked vascular response within the TG to two types of cutaneous trigeminal stimulation induced by punctuate application of a von Frey hair, we used animals implanted with a chronic window. Such a preparation allowed us to use a light anesthesia (isoflurane) to image this response. Despite previous demonstrations of a somatotopy of afferents and responsive neurons within the TG^38,43^, to the best of our knowledge, a consensual atlas of these subdivisions in three dimensions does not exist. Therefore, in order to image the areas responsive to the ophthalmic (V1) and maxillary (V2) TG division, we used sagittal planes at two different lateralities (L = 6 mm, Rotation 8° for V1 stimulation, Fig. 3a–e; L = 5 mm, Rotation 12° for V2, Fig. 3f–j). At these specific coordinates, we observed that application of punctuate static cutaneous stimulations using a 15 g von Frey hair, which is known to be nociceptive, induced a strong and reproducible BV increase in the ipsilateral TG. Interestingly, the activated TG area was different for V1 and V2 territories: a small, but deeply localized area for the ophthalmic branch (Fig. 3c, e, 12.0% increase, *p* = 3 × 10^−14^), while the area responsive to the maxillary branch was localized superficially (Fig. 3h, j, 15.9% increase, *p* = 3 × 10^−10^). These specific spatial clusters of hyperaemia within the TG conform with the known natural anatomical somatotopy of trigeminal neurons innervating V1 and V2 territories. Supplementary Figure 3 shows the modest reduction of delta BV during the stimulations in the unresponsive areas of the TG (dorsal for V1 and caudal for V2 experiments).

### Ultrasound localization microscopy (ULM) imaging reveals vascular features of the trigeminal blood flow

**Fig. 4:**
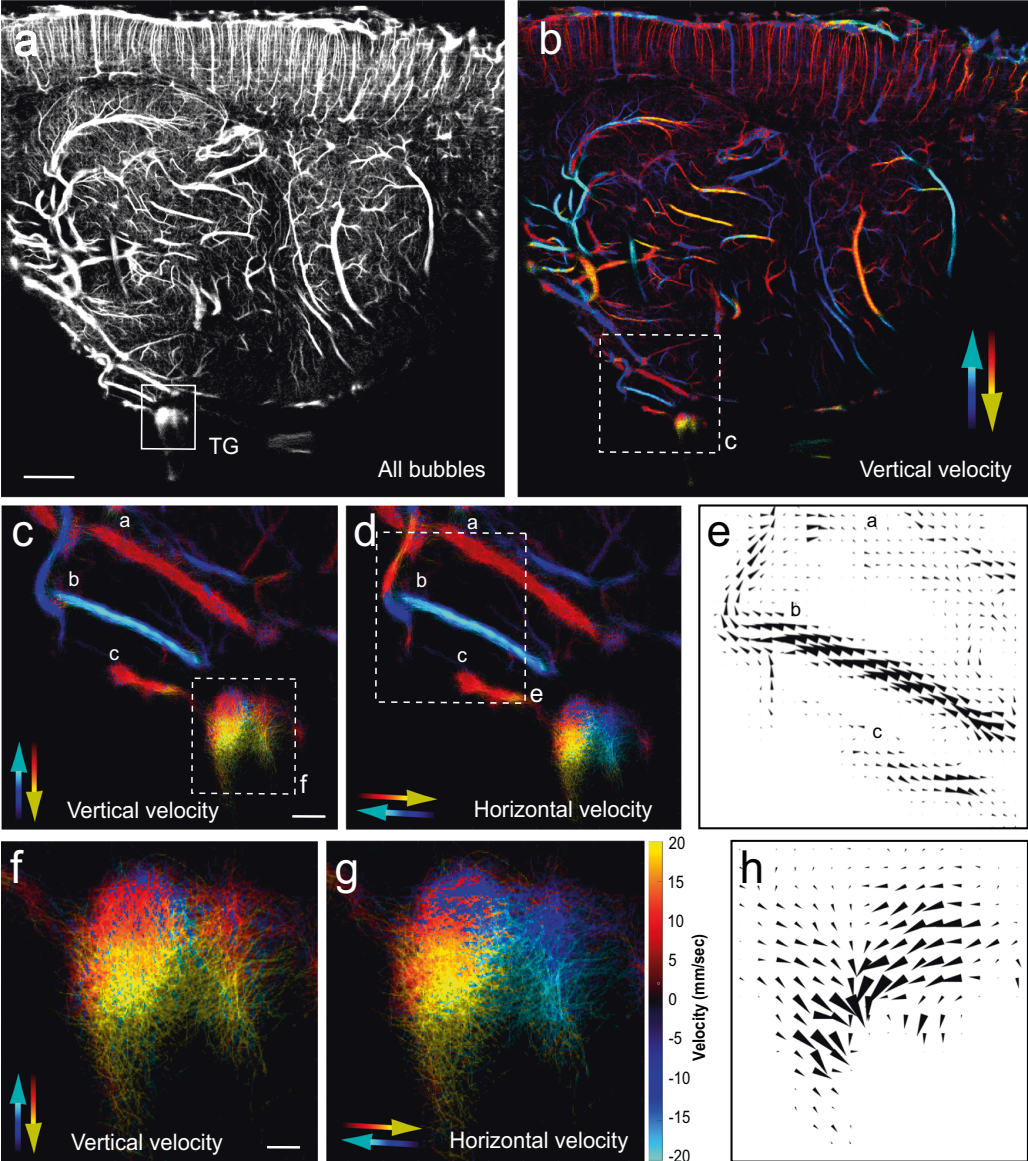
Ultrasound localization microscopy imaging (ULM) reveals the strong density and high speed of blood flow in the rat trigeminal ganglion in vivo. Following intravenous injection of bio-compatible microbubbles, known as conventional ultrasonic contrast agent (**a**), allows the determination of bubble velocity in both the vertical (**b**, **c**, **f**), or horizontal directions (**d**, **g**) through the analysis of single bubble trajectories (**b**–**h**). Pictures illustrate ULM imaging on a sagittal plane at lateral = 4.2 mm. **a** Doppler image of all bubbles in both the brain and the TG. The TG is surrounded by a white square. **b** ULM imaging of the whole brain and TG depicting velocity in the vertical direction. Panels **c**, **d** are a higher power magnification of the area delimited in **b**, **c**, and **d** show major blood vessels approaching the TG, which contain two descending veins and one ascending artery. Panels **f**, **g** are higher power magnification of **c** and **d**, respectively. Panels **e**, **h** are the field vectors in these blood vessels (**e**) and the TG (**h**). These field vectors illustrate not only the dense vascular network detected in the TG and the high blood speed in these vessels (9–20 mm/sec), but also the multiple directions of blood flow within the TG’s sub-parts imaged. **e**, **h** The size of the arrows is proportional to the local speed. Scale bars = **a**, **b**: 2 mm, **c**–**d**: 250 μm, **e**: 150 µm, **f**, **g**: 100 μm, **h**: 77 μm. The color bar (**g** right) applies for panels **b**–**d** and **f**, **g**.

To assess novel information regarding the vascular characteristics of the blood supply within the TG (flow direction and velocity), we used ULM implemented on the same ultrafast ultrasound scanner. This method allowed the measurement of flow of microbubbles inside blood vessels, at a microscopic scale in anesthetized animals^30,44^. We were able to provide a refined description of the blood vessels architecture and the measure and direction of the blood flow in 2 dimension. Our data confirmed (i) that the TG is highly vascularized, with a dense network of tortuous blood vessels. (ii) The organization of vessels does not look like any vascularized cerebral structures in the sagittal plane imaged (Fig. 4c–e versus Fig. 4b). Interestingly, the direction of blood flow within the TG was irregular (Fig. 4f–h), with two large tendencies: a ventro-posterior flux for the caudal part (right in Fig. 4f–h) and the opposite on the rostral part (left, Fig. 4h). Another difference with other cerebral structures, was the heterogeneity of speed in each portion of the TG, with a gradient of speed raging from 9 to 20 mm/sec (at the top, versus in the ventral aspect of the structure, respectively). Interestingly, these velocities were as high as those measured in the local major blood vessels depicted: a, b, and c (Fig. 4c–e, versus velocity in the TG in Fig. 4f-g). Due to their large diameter (a: 24–30 μm, b: 22–24 μm, c: 24–31 μm), high velocity (a: 8 mm/sec, b: 19 mm/sec, c: 10 mm/sec) and flow direction, we hypothesize that a and c are veins running in the ventro-anterior direction, while b is an artery^45^, running in the opposite direction (Fig. 4c–e).

## Discussion

Understanding the mechanisms underlying neurovascular coupling has been an active focus of research for a decade, as it is the cornerstone for the interpretation of functional MRI signals. This mechanism is also fundamental to the study of brain function and dysfunction in neurodegenerative diseases. The mechanistic studies undertaken were performed mostly at the level of the cerebral cortex and the olfactory bulb, and used a variety of complementary approaches, often in combination (see for review ref. ^46^), proposing an integrated approach of the cellular and molecular mechanisms involved in the neurovascular unit^23,47^. In contrast, much is still to be understood in the mechanism underlying the vascular response and the particular vascularization of the TG, as well as of the latter in both in health and disease. Functional imaging studies of the TG in animal models are crucially needed to understand debilitating trigeminal pathologies (such as migraine, corneal pain, or trigeminal neuralgia), whose prevalence increases since decades and remains a therapeutic challenge^48^.

However, such investigations are technically difficult to perform due to the small size of the TG and to its deep location. Here, we provide the first proof of concept of in-depth high-resolution TG imaging of vascular vector fields and speed of flow in naïve anesthetized rats. To date, a fundamental question in fUS imaging remains the number of activated neurons required to detect a subtle blood volume change. Here, we give for the first-time preliminary answers to this open question by showing that fUS imaging is sensitive enough to detect the functional activation of trigeminal nociceptors known to represent a very small number of neurons (~300 for the cornea).

This study aimed at providing a proof of concept for the hemodynamic responses in the TG following corneal nociceptive activation. We postulated that corneal stimulations and imaging of the TG in anesthetized animals constitutes a highly interesting model because corneal nociceptors (C and A delta nerve fibers) can be easily activated by mechanical stimulations^32,49^ and by the chemical stimulation of the TRPV1 receptor using capsaicin^33^. The ability to detect clear hemodynamic changes in response to corneal stimulations was a particular challenge in this study due to the small number of neurons innervating the cornea^38^.

Despite these challenges, our study evidences significant real-time (400 ms) hemodynamic responses of the TG to repeated sequences of C and A delta fiber mechanical activations (Fig. 2b–h). This ipsilateral activation, located in the V1 branch of the TG, is consistent with the known anatomy of the TG in rats^50^, and with the somatotopy of corneal afferents within the TG^38,43^. We went further in our investigations by evaluating the hemodynamic changes following the activation of polymodal nociceptors by the topical application of capsaicin, a TRPV1 agonist. We observed a strong phasic hemodynamic response in the ophthalmic division (Fig. 2i–l). The phasic aspect of this response is thought to be related both to difficulty to perform block design experiments, and also to the potent action of capsaicin known to strongly activate and sensitize the sensory afferents^1,7^.

While the existence of a somatotopy in the TG has long been suspected and is now confirmed by anatomical approaches in rodents^38,43^, only one neuroimaging study confirmed this concept in human subjects. Using fMRI, Borsook and collaborators demonstrated elegantly a somatotopic segregation of activated area in the human TG induced by either innocuous mechanical stimulations (brush) or noxious heat study applied on the face^2^. As this question was never addressed in preclinical models, we tested this hypothesis using chronically implanted animals and under isoflurane anesthesia. Both were necessary to image with a low level of anesthesia and no analgesia. Our results provided localized hemodynamic responses in the TG induced by punctuate cutaneous stimulations of the ophthalmic and maxillary divisions, using von Frey hairs. Importantly, the stimulation in these areas neither evoked responses in the same imaging plane, nor did it at the location within the TG (be it the dorsal or ventral part of the TG, see Fig. 3), suggesting, as observed in humans, a somatotopically defined area of the TG with responsive neurons. In conclusion, we demonstrate here for the first time a functional somatotopy within the TG of rats.

Until recently^51^, the vascularization of the TG has been studied and described mostly in human subjects^52^. Here we precisely localized the TG in rats using 2D or 3D fUS imaging and noted a high signal of BV, demonstrating that TG is richly vascularized. In toto staining of the vasculature (DiI experiments, Fig. 1f, g) and ULM in vivo, (Fig. 4) confirmed that the rat’s TG is richly vascularized, with highly tortuous vessels, especially in the CBRA. These morphological features are in agreement with the observations of Jimenez-Andrade et al., who reported a seven-times denser network of vascularization in the CBRA, compared to the nerve fibers rich area (NFRA) within the TG^53^. Interestingly, a similarly striking regional difference in the expression of tight junction proteins and the presence of functional blood–brain barrier (BBB) in the NFRA of the dorsal root ganglion (DRG), but not in the CBRA of the DRG has been reported^54^. Indeed, blood vessels that vascularize the CBRA have large fenestrations as compared to peripheral nerves, see for review refs. ^53,55^. Some studies exploring the permeability of the BBB showed that injected macromolecules are kept in the blood vessels in the NFRA, while they leak out from the blood vessels in the CBRA^56^. Therefore, there is an important dichotomy in the nature of the vascularization in the TG (and DRG) ganglia: while the NFRA has a minor vascularization and is well protected by the BBB, the CBRA has a dense vascularization, with a large lack of BBB. In addition to the high metabolic demand at the level of the cell bodies, these elements are likely the fundamental elements underlying the strong hemodynamic response to orofacial stimulations observed in the rats of our study and in human subjects.

Trigeminal nociceptive nerve fibers are known to express vasoactive neuropeptides, including CGRP and substance P^57^, that are released upon stimulation causing vasodilation that results in perivascular changes. CGRP is considered as the strongest vasodilating neuropeptide in human, and also participates in the sensitization of the trigemino-vascular system, in the peripheral sensitization and in hyperalgesia^58^. CGRP is expressed by 50% of the corneal neurons, 50% of them also co-expressing TRPV1^59^. Following these lines of evidence, the stimulation of corneal nociceptors through both mechanical stimulation and TRPV1 activation by capsaicin application is likely to have induced the release of CGRP. This release could be both local to the TG through a paracrine mechanism, as previously demonstrated^43^, and central (in the trigeminal brainstem sensory complex^58^). Once released, CGRP can signal on neighboring neurons, satellite and endothelial cells, glia, and mast cells, leading to vasodilatation^37^. Interestingly, a previous preclinical study showed that, while retrogradely traced neurons are localized in clusters in the TG (branches V1, V2, and V3 being separated from each other), capsaicin induces a spread of the tracer in all branches of the TG in the hours following injection in the temporomandibular joint. The tracer was not restricted anymore to neuronal soma, but was detected in satellite cells^43^. This phenomenon of cross-excitation within the entire TG induced by capsaicin is thought to be due to paracrine neuro-glial communication that leads to communication via gap junctions^43^. We propose that these mechanisms, previously hypothesized to be involved in orofacial sensitization and hypersensitivity^43^, may also be involved in the TG’s hemodynamic response increase quantified in this work and in previous clinical studies^4^.

In conclusion, this study constitutes the first demonstration of functional mapping of the hemodynamic response in the rodent TG evoked by natural trigeminal stimulations, and provides a new experimental approach to investigate the vascular features (blood flow and morphology) and the neurovascular coupling under physiological and pathophysiological pain conditions. Future investigations will be useful for the development/monitoring of therapeutical strategies for debilitating trigeminal chronic pain conditions.

## Methods

### Animals

All experiments were performed in compliance with the European Community Council Directive of September 22, 2010 (010/63/UE) and the local ethics committee (*Comité d’éthique en matière d’expérimentation animale* N°*59,‘Paris Centre et Sud’, project 2018-05 and 2018-19*). Accordingly, the number of animals in our study was kept to the necessary minimum. Experiments were performed on 28 male Sprague–Dawley rats (Janvier Labs; Le Genest St Isle, France), weighing 325–350 g at the beginning of the experiments. Animals (two per cage) arrived in the laboratory 1 week before the beginning of the experiment and were maintained under controlled conditions (22 ± 1 °C, 60 ± 10% relative humidity, 12/12 h light/dark cycle, food and water ad libitum).

### In toto labelling of the trigeminal vasculature using intracardiac perfusion of DiI

Labelling of the trigeminal vasculature was performed by intracardiac perfusion of the lipophilic fluorescent dye 1,1′-dioctadecyl-3,3,3′,3′-tetramethylindocarbocyanine perchlorate (DiI), a lipophilic carbocyanine dye, which incorporates into endothelial cell membranes upon contact, following a well-established protocol^60^. Three Sprague–Dawley rats were deeply anesthetized with an intraperitoneal (IP) injection of sodium pentobarbital (Dolethal, Ceva Santé, France, 150 mg.kg^−1^). Then, a thoracotomy was performed and an incision in the right atrium was made. Animals were perfused with 2 mL of saline solution (0.9% NaCl), followed immediately by 15 mL of DiI (0.012 mg.mL^−1^ Sigma–Aldrich, France) dissolved in 2% ethanol, 98% PBS (phosphate buffered saline solution, 0.02 mol.L^−1^), as previously described^60^, followed by 10 mL of paraformaldehyde 4% at the rate of 7 mL.min^−1^. Trigeminal ganglia were extracted and fixed overnight in 4% paraformaldehyde at 4 °C before cryoprotection in a 30% sucrose solution for 1 day. They were frozen in an OCT (Optimal Cutting Temperature) matrix in cooled isopentane (−40 °C) on dry ice, cut in 12 µm slices on a cryostat (Leica CM 3050 S, Wetzlar, Germany) and mounted on Superfrost slides (Thermofisher scientific, Waltham, Massachusetts, USA). Note: these animals were previously used in a study on the vasculature of the spinal cord architecture^61^.

### Calcitonin gene related peptide (CGRP) immunohistochemistry in the trigeminal ganglion

The sections that contained the DiI staining (see above) were washed three times in 0.1 M PBS. They were incubated for 1 h in a blocking solution of 0.1 M PBS containing 3% normal donkey serum and 0.1% triton X-100, and then for 24 h with a primary antibody at 4 °C. The primary antibody used in this study was mouse anti-CGRP (Sigma–Aldrich: Lot #083M4785, 1:250). CGRP was revealed using Alexa Fluor 594-conjugated donkey anti-rabbit antibody (1:500; Invitrogen). Finally, the sections were cover slipped.

Microscopic observations: tissue sections were examined using a Zeiss M1 epifluorescence microscope (Axio ImagerM1; Carl Zeiss) equipped with a digital camera (C11440-42U30; Hamamatsu Photonics) and an image acquisition software (Zen; Carl Zeiss).

### Surgical procedures and preparation for imaging

As the skull attenuates and (possibly) disturbs the propagation of ultrasounds, the imaging of the brain and in particular of the trigeminal ganglion (that is located below the brain) requires the removal of the skull. In this study, experiments of functional ultrasound were performed either through an acutely prepared craniotomy (results presented in Figs. 1, 2, and 4) or through a chronically implanted window (Fig. 3). These windows were performed between Bregma +4.0 mm and Bregma −9.0 mm, allowing the scanning of a large part of the brain and the TG. During all the procedures, the animal’s body temperature was maintained at 37.0 °C using a heating pad (Physitemp, Clifton, USA). Heart and breathing frequencies were constantly measured using ECG, a spirometer from AD Instruments and the ‘Labchart’ software (AD Instruments, Paris, France).

#### Preparation of an acute craniotomy

Under deep anesthesia (intraperitoneal (IP) bolus of medetomidine (Domitor, 0.4 mg.kg^−1^) and ketamine (Imalgène, 40 mg.kg^−1^)), the animal was placed on a stereotaxic frame and a craniotomy (removal of the skull) was performed as previously described^19^ between Bregma and Lambda. Some ELMA cream (AstraZeneca, UK) was placed in the ear bars in order to prevent discharge from nociceptors at ear level.

#### Preparation of chronically implanted cranial windows

An antalgic preanesthetic treatment buprenorphine (0.05 mg/kg) was given 20 min before the initiation of anesthesia. Anesthesia was induced with 2% isoflurane (0.35 l/min air and 0.15 l/min d’O2 using Minerve apparatus (Esternay, France)). A subcutaneous injection of local anesthetic (Xylovet (4 mg/kg) was performed 20 min before skin incision. We excised the parietal and frontal flaps by drilling and gently moving the bone away from the dura mater. The opening exposed the brain from Bregma +4.0 to Bregma −9.0 mm, with a maximal width of 14 mm. A prosthetic skull was sealed in place with acrylic resin (GC Unifast TRAD), and the residual space was filled with saline solution. The prosthetic skull is composed of polymethylpentene (Goodfellow, Huntington UK, goodfellow.com), a standard biopolymer used for implants^15,25^. This material has tissue-like acoustic impedance that allows undistorted propagation of ultrasound waves at the acoustic gel-prosthesis and prosthesis-saline interfaces. The prosthesis was cut out of a film of 250 µm thickness and permanently sealed to the skull. Particular care was taken not to tear apart the dura to prevent cerebral damage. Animals were subcutaneously injected with anti-inflammatory drug (Metacam, 0.2 mg/kg, once a day for 7 days) and prophylactic antibiotics (Borgal, 16 mg/kg once a day for 7 days), and postoperative care was performed for 7 days. Animals recovered quickly and were used for data acquisition after a conservative 10-day resting period.

#### Imaging sessions

Two milliliters of saline solution were gently dropped on the brain, followed by echographic gel (Dexco Médical, France). The ultrasonic probe was then positioned just above the window using a 4-axis motorized system on which the ultrasound probe was fixed^19^.

For animals with an acute window, as previously described^19,61^, 45 min after induction (when the craniotomy was finished), the anesthesia was maintained but reduced, with a subcutaneous perfusion of medetomidine (0.1 mg/kg/h) and ketamine (12.5 mg/kg/h) using a syringe pump. As previously observed^61^, it was preferable to wait in order to reach stable physiological parameters and a reproducible level of anesthesia (respiratory frequency around 80–90 rpm).

For animals with a chronic window, the anesthesia was induced with 3% isoflurane (0.35 l/min air and 0.15 l/min d’O_2_ using Minerve apparatus (Esternay, France)). Once the animal was installed on the stereotaxic frame, the level of isoflurane was gradually reduced to 1–0.75% over 30–40 min. Functional stimulations of the V1 or V2 territories were performed using 1–0.75% isoflurane, at a respiratory frequency of 80–90 rpm.

Each imaging session lasted from 3 to 4 h.

##### 2D and 3D imaging scans of the brain and TG

In all animals on which we performed fUS experiments, a linear scan was first performed with a few successive antero-posterior 2D scans at (100 × 100 × 400 μm^3^) resolution in order to reproducibly recognize the imaging plane of interest containing the TG. The spatial extent of the TG was measured and the probe was positioned in the desired plane of imaging (Bregma + 4.56 mm, see supplementary Fig. 1). This plane corresponds to the medio-anterior part of the TG, where the neurons of branch V1 are expected to be found^50^.

Using a prototype ultrasonic ultrafast neuroimager (Iconeus, Paris, France), in *N* = 2 animals, a 3D-scan of the entire window (Bregma to Lambda) was performed with a tomographic approach. This approach based on the acquisition of several linear scans with different orientations of the probe enables the reconstruction of 3D angiography with isotropic voxel resolution (100 × 100 × 100 μm^3^) using a linear ultrasonic probe^62^. A total of 19 probe orientations of the probe were used and, for each orientation, 81 successive 2D scans were acquired with 0.2 mm steps. The isotropic 3D volume reconstructed (IcoStudio, Iconeus, Paris, France) is shown in Supplementary Movie 1 and Fig. 2b.

##### Peripheral stimulations

**Internal positive control: stimulation of the left whisker pad**: To check the adequacy of the level of anesthesia, we performed a routine test in each experiment, which consisted in measuring the evoked haemodynamic response in the barrel cortex (S1BF) induced by stimulation of the ipsilateral whisker pad (Supplementary Fig. 1), as previously performed in our seminal article^18^. A train of six stimulations at 4 Hz lasting 20 sec, separated by 20 sec, were performed using an Arduino. In the rare cases (2 over 30) where this test did not elicit any response after multiple retests in various planes of the barrel cortex, the animal was euthanized and excluded from the experiment.

**Mechanical and chemical corneal stimulations**: Mechanical stimulations consisted of six manual stimulations of the cornea using a soft brush (gentle stroke, see Supplementary Movie 2). The stimulations lasted 10 sec, separated by 20 sec, with a resting time of 30 sec before and after these repetitions.

Chemical stimulations were performed using topical corneal application of capsaicin (10 μM, Sigma–Aldrich). After 60 sec in absence of any stimulation (baseline), a 2 × 2 mm filter paper impregnated with 50 μL of 10 μM capsaicin was applied on the cornea for 3 min. The cornea was then washed using a saline solution.

**Static mechanical stimulations of the ophthalmic and maxillary territories**: Four static mechanical stimulations were applied manually using a calibrated von Frey hair #15 (Anesthesiometer), which applies reproducibly a force of 17 g, which is known to be nociceptive in these territories in (awake) naïve animals. The stimulations lasted 5 sec, separated by 55 sec, with a resting time of 60 sec before and after these repetitions.

##### fUS imaging: sequences of imaging and signal processing

fUS imaging was performed using a linear ultrasound probe (128 elements, 15 MHz, 110 µm pitch, 8 mm elevation focus, Iconeus, Paris, France) driven by a prototype ultrasonic ultrafast neuroimager (Iconeus, Paris, France). The fUS imaging sequence operated as follows: the brain and TG were insonified by ten successive tilted plane waves with an angle varying from −10° to 10° with a 5.5 kHz Pulse Repetition Frequency (PRF). The backscattered echoes were recorded by the transducer array and beamformed to produce a block of 200 consecutive ultrafast images with a framerate of 500 Hz. In order to filter the Blood Volume (BV) and to remove the tissue signal, we used a clutter filter based on Singular Value Decomposition (SVD) applied to 200 successive frames^63^ by removing the 60 first singular vectors which correspond mainly to the tissue space. Finally, a Power Doppler image was obtained by integrating the energy of the filtered frames, resulting in a Power Doppler image every 400 ms.

**Doppler signal analysis and activation maps**: Doppler data were analyzed using a generalized linear model approach (GLM) implemented in Matlab^61^ in order to obtain the Z-score and *p*-value maps. Here, the stimulus response was modeled by the convolution of the stimulus paradigm with a four half-cosines canonical hemodynamic response function^64^. The parameters were adjusted in preliminary experiments by nonlinear fitting (fmincon, Matlab) of the model output to the measured BV response. The activation maps show the Z-score of all significant pixels after Bonferroni correction in the image (corresponding to a *p*-value < 0.05 before Bonferroni correction).

We drew the ipsilateral region of interest (ROI) around the activation area thanks to the thresholded Z-score map and the contralateral ROI was drawn by symmetry. The two signals were averaged along the two spatial dimensions in order to obtain a single temporal signal. The signal was then expressed as a BV (Blood Volume) relative difference (in percent), or ∆BV, by subtracting the BV baseline (calculated for each acquisition by averaging all the temporal data within the TG where the stimulation pattern was strictly equal to 0) and by dividing by the BV baseline. Mean ∆BV values over time were computed at baseline, i.e., during the periods without chemical or mechanical stimulation of the cornea, and during stimulation, i.e., during the periods with stimulation, and are denoted by ∆BV_BL_ and ∆BV_STIM_.

**Statistical analysis of the evoked trigeminal hemodynamic responses**: The statistical analysis was performed using Matlab Version 9.7.0.1261785 (R2019b). The data were modelled using linear mixed models (LMM), which are suited to the case of nonindependent, hierarchical data (∆BV values from several acquisitions, in several animals), and of factors of interest having fixed effects (ipsilateral vs contralateral TG, baseline vs stimulation) as well as random effect factors (acquisition, animal).

In the case of the corneal stimulation, our aim was to establish the significance of the difference between ipsilateral and contralateral ∆BV values during stimulation (Fig. 2), with a single stimulation per acquisition, and possibly several acquisitions per rat. The LMM was hence of the form Y_ik_ = δ + R_i_ + W_ik_, where Y^ik^ denotes the difference between ipsilateral and contralateral ∆BV_STIM_ values for rat i and stimulation k, δ denotes the fixed effect of the side, the zero mean R_i_ models the random rat effect, and W_ik_ is the residual error term.

In the case of the mechanical stimulation, our main aim was to establish the significance of the difference between ipsilateral and contralateral ∆BV values during stimulation, with six stimulations per acquisition, and possibly several acquisitions per rat. The LMM was hence of the form Y_ijk_ = δ + R_i_ + A_ij_ + W_ijk_, where Y^ijk^ denotes the difference between ipsilateral and contralateral ∆BV_STIM_ values for rat i, acquisition j, and stimulation k, δ denotes the fixed effect of the side, the zero mean R_i_ models the random rat effect, the zero mean A_ij_ models the random acquisition effect, and W_ijk_ is the residual error term.

The same model structure was also used to compare stimulation to baseline ∆BV values, either on the ipsilateral TG (Y = ∆BV_STIM_^ipsi^ − ∆BV_BL_^ipsi^), or on the contralateral TG (Y = ∆BV_STIM_^contra^ − ∆BV_BL_^contra^) (see Figs. 2 and 3).

∆BV values were first transformed by taking their square root (i.e., √∆BV if ∆SB ≥ 0, √−∆BV if ∆BV < 0) to homogenize their variance and normalize their distributions. The LMMs were fitted with the restricted maximum likelihood method using Matlab’s function *fitlme*. The normality of the residuals was checked with Shapiro-Wilk’s test. The test of nullity the fixed effect factor was performed with an F test using Matlab’s function *fixedEffects*. The significance of the random effects was evaluated with a likelihood ratio test using Matlab’s function *compare*.

##### Ultrasound localization microscopy (ULM)

Three animals were used for this procedure. A catheter filled with saline solution was inserted in the rat jugular vein before the positioning of the animal on the stereotaxic frame. ULM was performed similarly to the methods described in ref. ^31^, but using continuous injections of Sonovue (Braco, Italy) reconstructed in 5 mL of saline at the rate of 3.5 mL/h. A total of 750 blocks composed of 400 compounded frames at a 1000 Hz framerate (with angles at −5°, −2°, 0°, +2°, +5°, PRF = 5000 Hz, 12 mm imaging depth) were acquired using the same system as above. The total acquisition lasted 300 s. Beamformed data were filtered using the SVD spatio-temporal filter described in ref. ^65^ and the 10 first singular values were removed to extract microbubbles signals from the surrounding tissues. Microbubbles were detected as the brightest local maxima in the images. The tracking of the maximal positions was performed using a classical particle tracking algorithm (simpletracker.m available on Mathworks (Matlab Central) ©Jean-Yves Tinevez, wrapping Matlab Munkres algorithm implementation of ©Yi Cao 2009). The successive positions gathered in one track were used to compute the interframe microbubble velocity vector components (along probe *x*-axis and depth *z*-axis) and absolute velocity magnitude. Density maps were computed by counting all the positions detected in one pixel during the acquisitions. Velocity maps were computed as the mean velocity of all MBs passing through one pixel during the whole acquisition. The pixel size for image reconstruction was ~6.5 µm.

Density maps were computed by counting all the positions detected in one pixel during the acquisitions. Velocity maps were computed as the mean velocity of all microbubbles passing through the pixel during the whole acquisition.

### Reporting summary

Further information on research design is available in the Nature Research Reporting Summary linked to this article.

## Supplementary information

Supplementary Information

Description of Additional Supplementary Files

Supplementary Movie 1

Supplementary Movie 2

Reporting Summary

## Data Availability

All data presented in this study are available in open access in Zenodo, at the following 10.5281/zenodo.6331619.

## Code availability

Custom codes used for the collection of fUS data are protected by INSERM and can only be shared upon reasonable request from the corresponding author, with the written agreement of INSERM.
