# Supplementary material for: Ultrasound localization microscopy and functional ultrasound imaging reveal atypical features of the trigeminal ganglion vasculature
Source: Commun Biol. 2022 Apr 7;5:330. doi: 10.1038/s42003-022-03273-4 (PMC8989975; doi:10.1038/s42003-022-03273-4)
Supplement: Supplementary file 1 — Supplementary Information [file 42003_2022_3273_MOESM1_ESM.pdf]

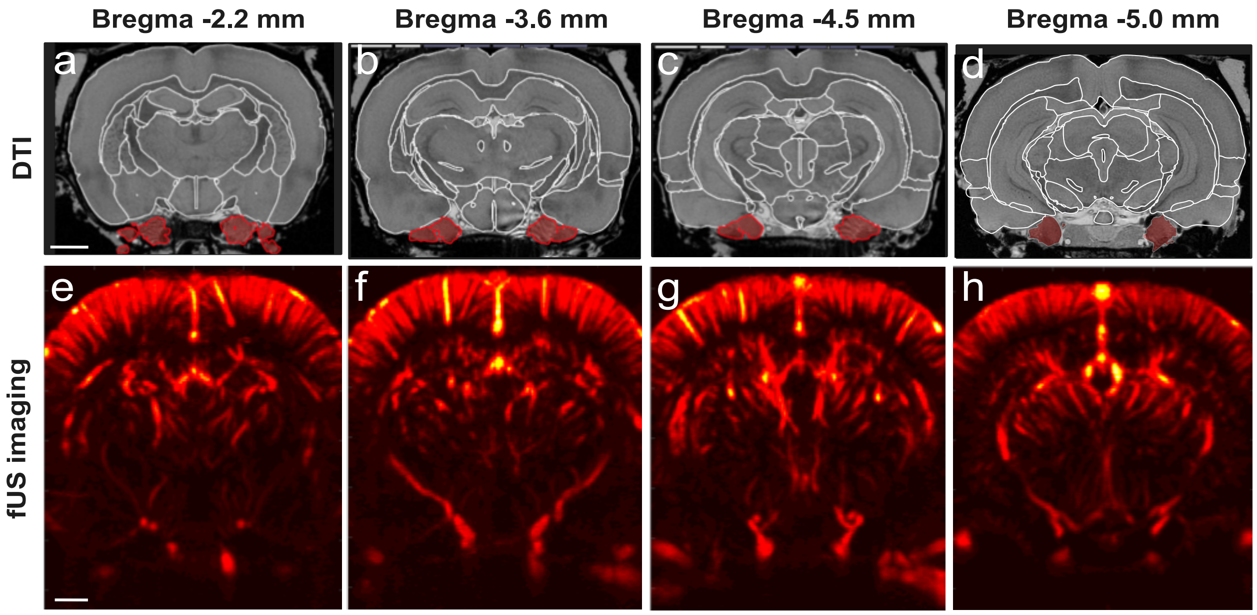

Supplementary figure 1:

Comparative localization of the trigeminal ganglion imaged using DTI (from Waxholm's tractography atlas i.e. defined using fiber tracks 34–36, a-d) and fUS (Ultrafast Doppler, e-h) at different antero-posterior coordinates in the rat. Images in a, which are distributed under the Creative Commons Attribute Non-Commercial Share-Alike license, were copied, without any modification from the following website: https://scalablebrainatlas.incf.org/rat/PLCJB14.

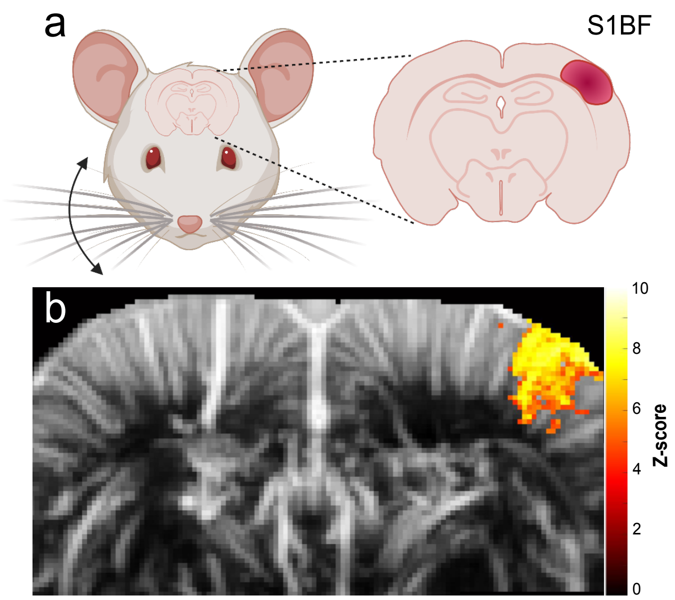

Supplementary figure 2:

Functional ultrasound imaging of the evoked hyperaemia in the S1BF induced by whisker pad ‘s stimulation. a: Ipsilateral stimulation of the right whisker pad known to induce a local hemodynamic response in the contralateral primary sensory cortex, barrel field (S1BF). Failure to observe such a response at the beginning of our experiments induced the exclusion of the animal from the protocol. b: Example of evoked responses, i.e. statistically significant increased positive pixels (significant Z-score) in a representative animal. The panel a was created with Biorender.com.

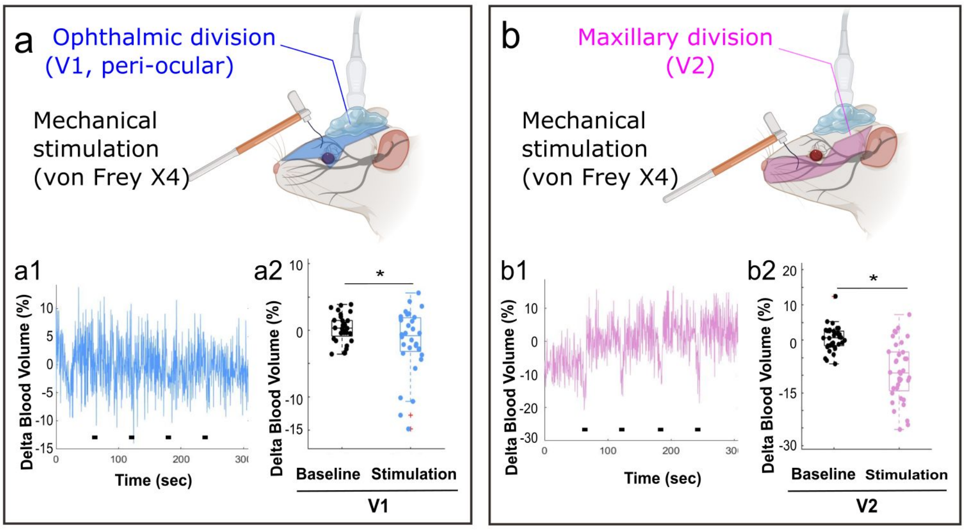

Supplementary figure 3:

Analysis of the changes of delta CBV in the part of the trigeminal ganglion that did not display a functional hyperemia induced by mechanical stimulation of the ophthalmic and maxillary areas. a-b top: Diagram representing the stimulated trigeminal subdivision (a: Ophthalmic, b: Maxillary) as a reminder of the experimental design (idem as figure 3). a1, b1 show the variation of delta CBV over time in the part in the TG that showed a lack of significant changes at the z-map in the examples presented in figure 3, i.e. the dorsal part in the V1 and ventral in the V2 experiments, respectively. a2-b2: quantification of the changes in delta BV in the aforementioned ROI for all animals and acquisitions (a2: N=30 stimulations in N= 3 animals; b2: N=36 stimulations in N=3 animals). These results suggest a modest but statistically significant reduction of CBV in the part of the TG away from the somatotopically activated area. * $P < 0.05$. Panels a-b were created with Biorender.com.
