# Supplementary material for: Ultrasound localization microscopy and functional ultrasound imaging reveal atypical features of the trigeminal ganglion vasculature
Source: Commun Biol. 2022 Apr 7;5:330. doi: 10.1038/s42003-022-03273-4 (PMC8989975; doi:10.1038/s42003-022-03273-4)
Supplement: Supplementary file 2 — Description of Additional Supplementary Files [file 42003_2022_3273_MOESM2_ESM.pdf]

Description of Additional Supplementary Files

File name: Supplementary Video 1
Description: Video showing 3- dimension tomographic scan performed in a rat (after removal of the skull to avoid attenuation). It shows the entire vasculature of the rat brain and TGs (below).

File name: Supplementary Video 2
Description: Video showing, in real time in a typical experiment (using Iconeus One imager and the software 'Neuroshop'), the increased blood volume in the trigeminal ganglion during the application of repeated corneal mechanical stimulations.
