# Supplementary material for: Ultrasound localization microscopy and functional ultrasound imaging reveal atypical features of the trigeminal ganglion vasculature
Source: Commun Biol. 2022 Apr 7;5:330. doi: 10.1038/s42003-022-03273-4 (PMC8989975; doi:10.1038/s42003-022-03273-4)
Supplement: Supplementary file 5 — Reporting Summary [file 42003_2022_3273_MOESM5_ESM.pdf]

nature portfolio

Corresponding author(s): S. Pezet

Last updated by author(s): Feb 13, 2022

Reporting Summary

Nature Portfolio wishes to improve the reproducibility of the work that we publish. This form provides structure for consistency and transparency in reporting. For further information on Nature Portfolio policies, see our Editorial Policies and the Editorial Policy Checklist.

Statistics

For all statistical analyses, confirm that the following items are present in the figure legend, table legend, main text, or Methods section.

n/a	Confirmed	
☐	☒	The exact sample size (n) for each experimental group/condition, given as a discrete number and unit of measurement
☐	☒	A statement on whether measurements were taken from distinct samples or whether the same sample was measured repeatedly
☐	☒	The statistical test(s) used AND whether they are one- or two-sided<br>*Only common tests should be described solely by name; describe more complex techniques in the Methods section.*
☐	☒	A description of all covariates tested
☐	☒	A description of any assumptions or corrections, such as tests of normality and adjustment for multiple comparisons
☐	☒	A full description of the statistical parameters including central tendency (e.g. means) or other basic estimates (e.g. regression coefficient) AND variation (e.g. standard deviation) or associated estimates of uncertainty (e.g. confidence intervals)
☐	☒	For null hypothesis testing, the test statistic (e.g. F, t, r) with confidence intervals, effect sizes, degrees of freedom and P value noted<br>*Give P values as exact values whenever suitable.*
☒	☐	For Bayesian analysis, information on the choice of priors and Markov chain Monte Carlo settings
☒	☐	For hierarchical and complex designs, identification of the appropriate level for tests and full reporting of outcomes
☒	☐	Estimates of effect sizes (e.g. Cohen's d, Pearson's r), indicating how they were calculated

Our web collection on statistics for biologists contains articles on many of the points above.

Software and code

Policy information about availability of computer code

Data collection	Custom codes used for the collection of fUS data are protected by INSERM and can only be shared upon request, with the written agreement of INSERM.
Data analysis	Data were analyzed on Matlab.

For manuscripts utilizing custom algorithms or software that are central to the research but not yet described in published literature, software must be made available to editors and reviewers. We strongly encourage code deposition in a community repository (e.g. GitHub). See the Nature Portfolio guidelines for submitting code & software for further information.

Data

Policy information about availability of data

All manuscripts must include a data availability statement. This statement should provide the following information, where applicable:

- Accession codes, unique identifiers, or web links for publicly available datasets
- A description of any restrictions on data availability
- For clinical datasets or third party data, please ensure that the statement adheres to our policy

Source data will be provided before publication of this article on a repository website.

Field-specific reporting

Please select the one below that is the best fit for your research. If you are not sure, read the appropriate sections before making your selection.

☒ Life sciences ☐ Behavioural & social sciences ☐ Ecological, evolutionary & environmental sciences

For a reference copy of the document with all sections, see nature.com/documents/nr-reporting-summary-flat.pdf

Life sciences study design

All studies must disclose on these points even when the disclosure is negative.

Sample size	Our study describes a new neuroimaging modality in the trigeminal ganglion. The first part of the study is rather a proof of concept study, that required N=3 per type of experiment (minimal to be conclusive). In the following steps, we used the variability of the technique to evaluate the number of animals/ stimulations to be performed to observe a change. However, these calculations could only be indicative, as we had never imaged in this particular organ. Finally, we used a linear model to analyse the effects obtained.
Data exclusions	As described in the materials and methods, we included a positive control at the beginning of the experiments (fonctional activation of the whisker that is know to induce an hemodynamic response in the contralateral barrel field). All animals that did not display such activations were excluded (2 out of 30).
Replication	The LMM model shows that the measures were reproducible. WE also performed internal tets to show that the spatial actibvation maps were reproducible intra and inter-animals.
Randomization	We did not need to do any randomization in this study.
Blinding	Our study does not compare experimental groups. Therefore, there was no need for blinding.

Reporting for specific materials, systems and methods

We require information from authors about some types of materials, experimental systems and methods used in many studies. Here, indicate whether each material, system or method listed is relevant to your study. If you are not sure if a list item applies to your research, read the appropriate section before selecting a response.

Materials & experimental systems

n/a	Involved in the study	
☒	☐	Antibodies
☒	☐	Eukaryotic cell lines
☒	☐	Palaeontology and archaeology
☐	☒	Animals and other organisms
☒	☐	Human research participants
☒	☐	Clinical data
☒	☐	Dual use research of concern

Methods

n/a	Involved in the study	
☒	☐	ChIP-seq
☒	☐	Flow cytometry
☒	☐	MRI-based neuroimaging

Animals and other organisms

Policy information about studies involving animals; ARRIVE guidelines recommended for reporting animal research

Laboratory animals	Adult male Sprague Dawley rats
Wild animals	This study did not include wild animals.
Field-collected samples	N/A
Ethics oversight	This project was approved by the the local ethics committee (Comité d'éthique en matière d'expérimentation animale N°59,'Paris Centre et Sud', project 2018-05 and 2018-19).

Note that full information on the approval of the study protocol must also be provided in the manuscript.
